# Supplementary figures and images for: Continuous sensing of IFNα by hepatic endothelial cells shapes a vascular antimetastatic barrier
Source: eLife. 2022 Oct 25;11:e80690. doi: 10.7554/eLife.80690 (PMC9596162; doi:10.7554/eLife.80690)

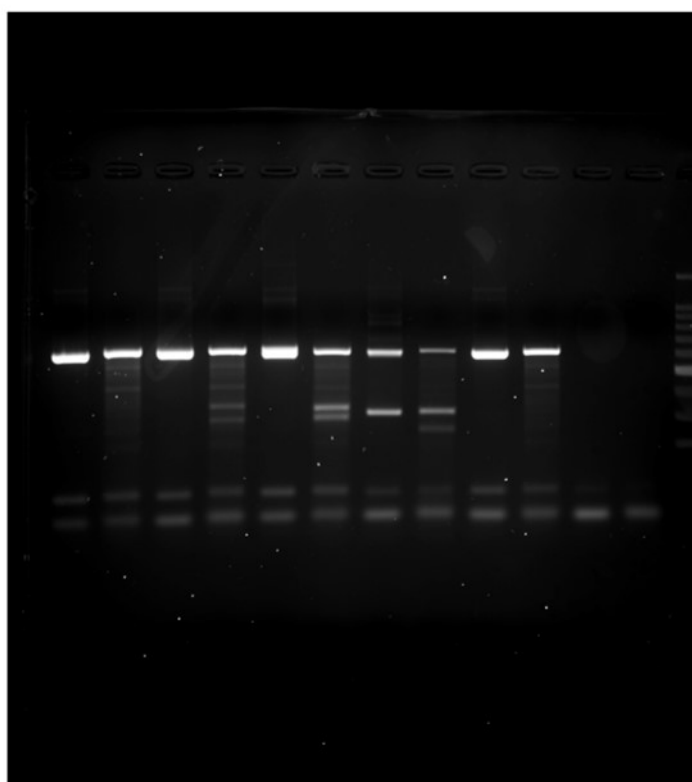

Supplement: Figure 4—figure supplement 1—source data 1. [file elife-80690-fig4-figsupp1-data1.zip › Figure 4 - figure supplement 1 - source data 1/Figure 4 - figure supplement 1_Source data 1.pdf]

Figure 4 - figure supplement 1-source data 1

Uncropped gel with labeling of panel (A)

A

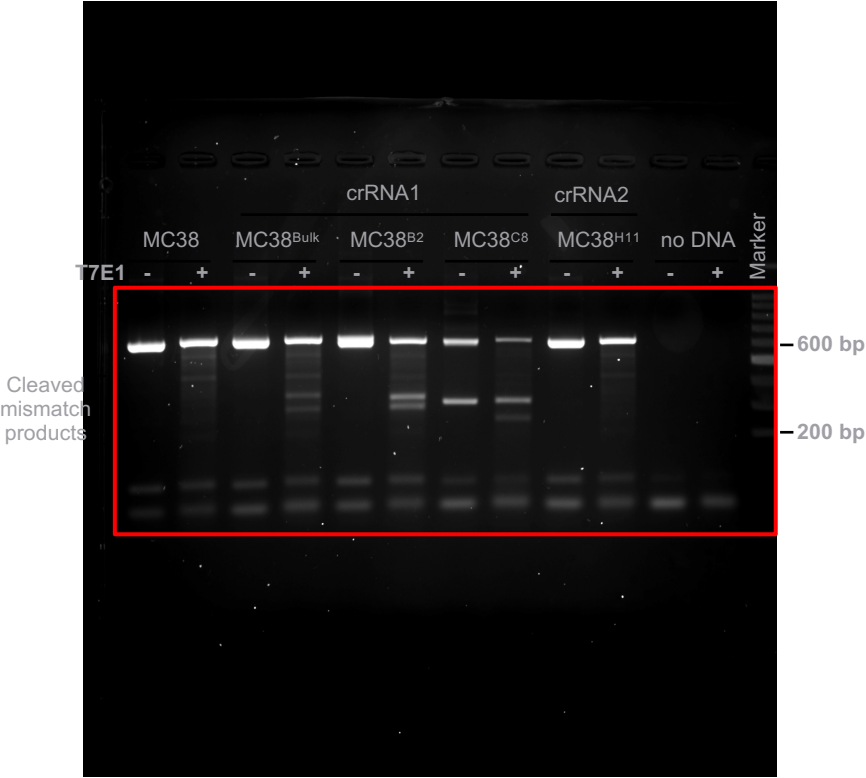

Supplement: Figure 4—figure supplement 1—source data 1. [file elife-80690-fig4-figsupp1-data1.zip › Figure 4 - figure supplement 1 - source data 1/Figure 4 - figure supplement 1_Source data 1_uncropped.pdf]

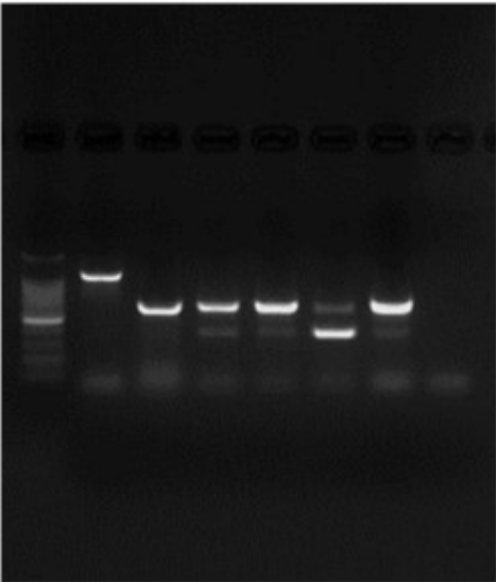

Supplement: Figure 4—figure supplement 1—source data 3. [file elife-80690-fig4-figsupp1-data3.zip › Figure 4 - figure supplement 1 - source data 3/Figure 4 - figure supplement 1_Source data 3.pdf]

Uncropped gel with labeling of panel (D)

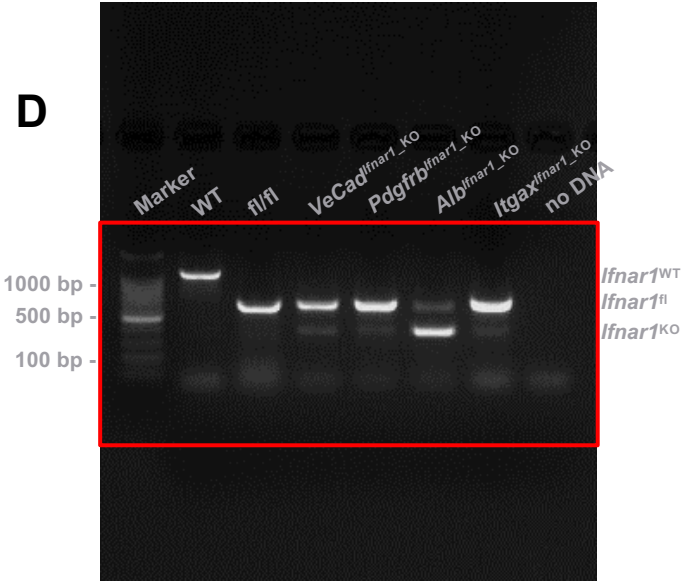

Supplement: Figure 4—figure supplement 1—source data 3. [file elife-80690-fig4-figsupp1-data3.zip › Figure 4 - figure supplement 1 - source data 3/Figure 4 - figure supplement 1_Source data 3 uncropped.pdf]

*Ifnar1<sup>fl/fl</sup>**VeCad<sup>Ifnar1\_KO</sup>*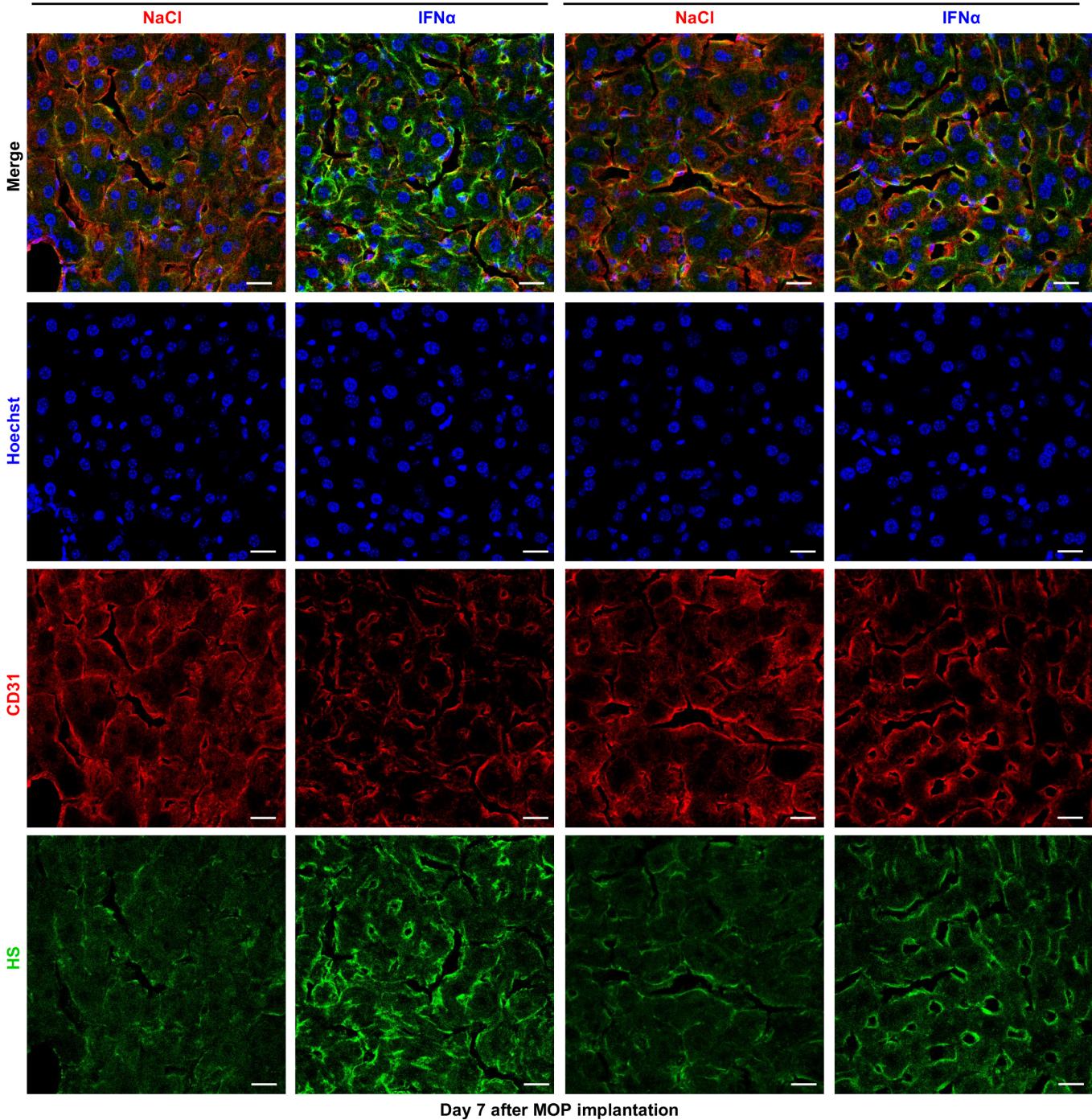

Supplement: Figure 5—source data 2. [file elife-80690-fig5-data2.zip › Figure 5 - Source data 2/Figure 5 - Source data 2.pdf]

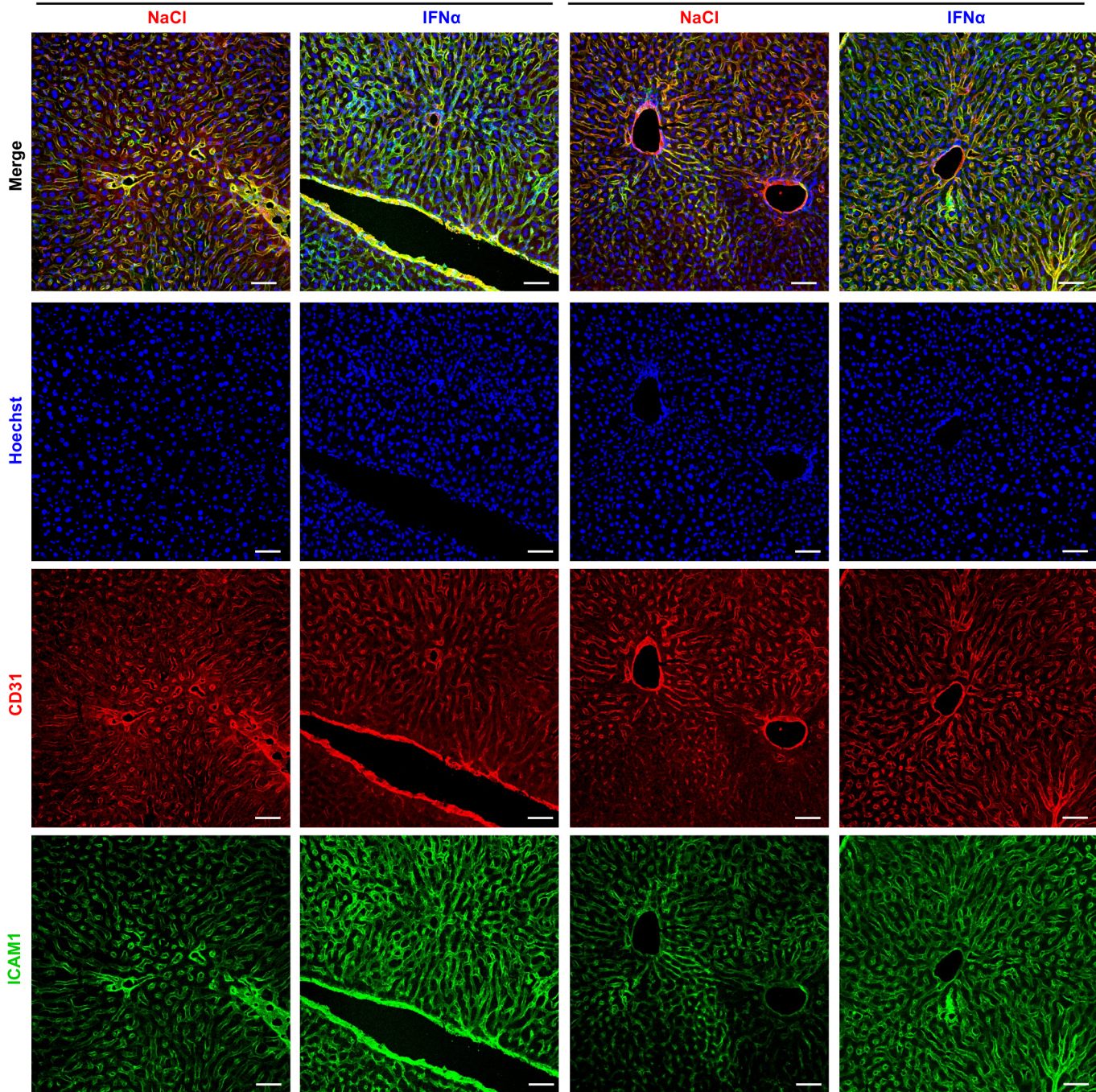

Supplement: Figure 5—figure supplement 1—source data 4. — High-magnification immunofluorescence images of each channel. [file elife-80690-fig5-figsupp1-data4.zip › Figure 5 - figure supplement 1 - source data 4/Figure 5 - figure supplement 1 - source data 4.pdf]
