# Supplementary material for: Continuous sensing of IFNα by hepatic endothelial cells shapes a vascular antimetastatic barrier
Source: eLife. 2022 Oct 25;11:e80690. doi: 10.7554/eLife.80690 (PMC9596162; doi:10.7554/eLife.80690)
Supplement: Figure 5—figure supplement 1—source data 3. — High-magnification immunofluorescence images of each channel. [file elife-80690-fig5-figsupp1-data3.zip › Figure 5 - figure supplement 1 - source data 3/Figure 5 - figure supplement 1 - source data 3.pdf]

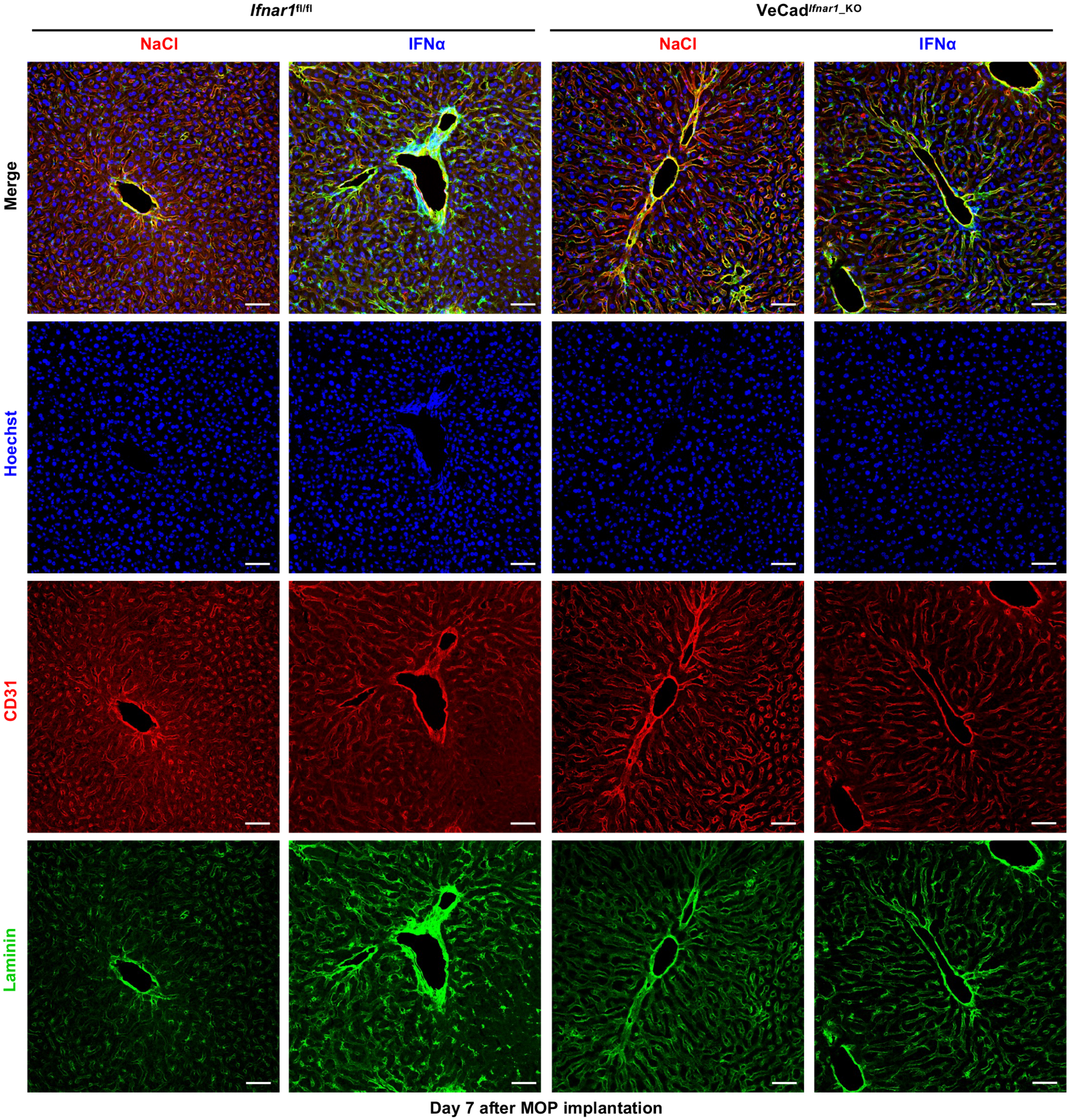
*Ifnar1*fl/fl
VeCad*Ifnar1*_KO
NaCl
IFNα
NaCl
IFNα
Merge
Hoechst
CD31
Laminin
Day 7 after MOP implantation
