# Supplementary material for: Continuous sensing of IFNα by hepatic endothelial cells shapes a vascular antimetastatic barrier
Source: eLife. 2022 Oct 25;11:e80690. doi: 10.7554/eLife.80690 (PMC9596162; doi:10.7554/eLife.80690)
Supplement: Figure 5—figure supplement 2—source data 1. — High-magnification immunofluorescence images of each channel. [file elife-80690-fig5-figsupp2-data1.zip › Figure 5 - figure supplement 2 - source data 1/Figure 5 - figure supplement 2 - source data 1.pdf]

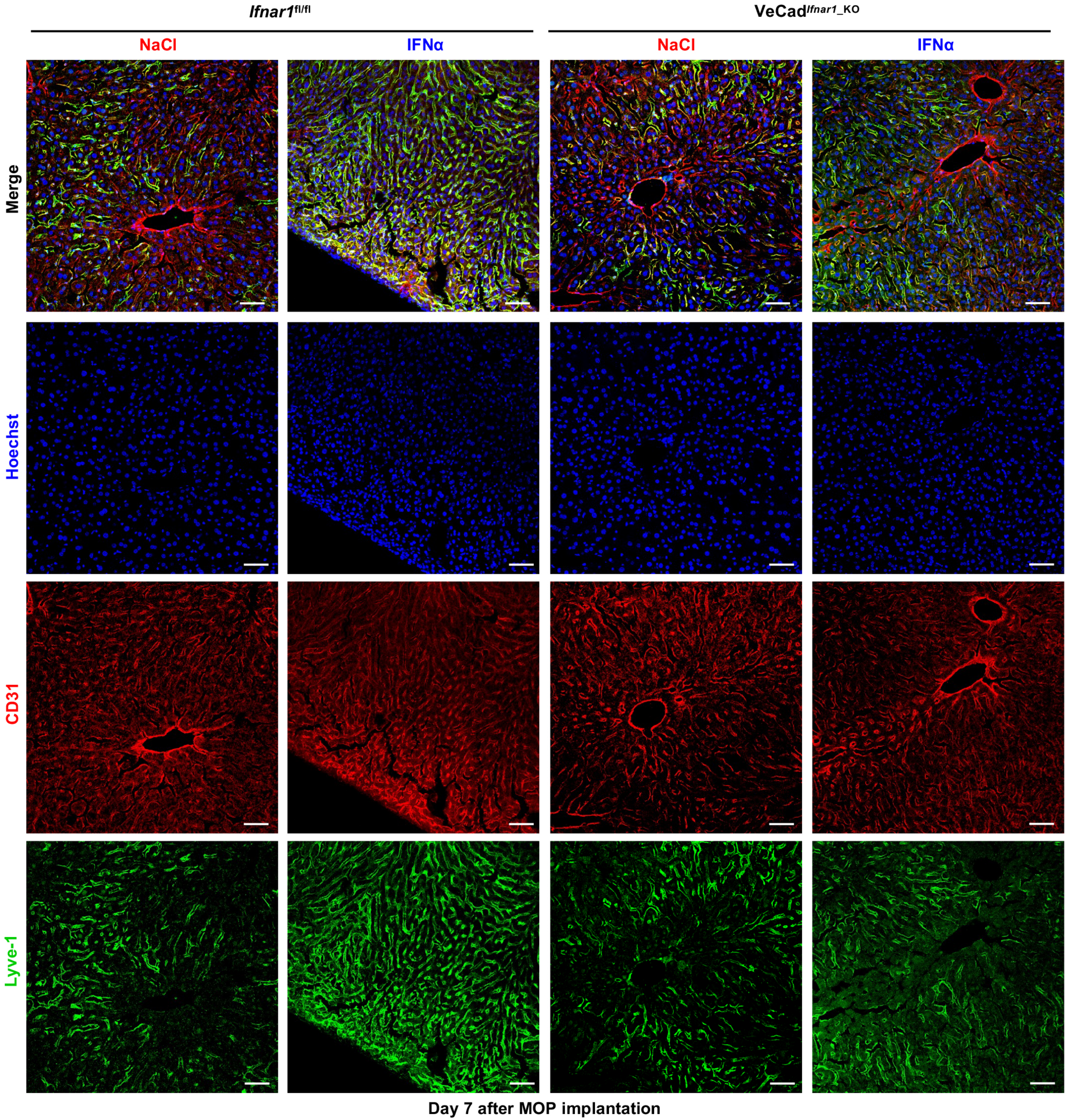
Ifnar1fl/fl
VeCadIfnar1_KO
NaCl
IFNα
NaCl
IFNα
Merge
Hoechst
CD31
Lyve-1
Day 7 after MOP implantation
